# Ultraflexible Liposome Nanocargo as a Dermal and Transdermal Drug Delivery System

**DOI:** 10.3390/nano11102557

**Published:** 2021-09-29

**Authors:** Kalvatala Sudhakar, Shivkanya Fuloria, Vetriselvan Subramaniyan, Kathiresan V. Sathasivam, Abul Kalam Azad, Shasank S. Swain, Mahendran Sekar, Sundram Karupiah, Omji Porwal, Alaka Sahoo, Dhanalekshmi Unnikrishnan Meenakshi, Vipin Kumar Sharma, Sanjay Jain, R. Narayana Charyulu, Neeraj Kumar Fuloria

**Affiliations:** 1 School of Pharmaceutical Sciences (LIT-Pharmacy), Lovely Professional University, Jalandhar 144411, India; ckbhaipharma@gmail.com; 2 Faculty of Pharmacy, AIMST University, Bedong 08100, Kedah, Malaysia; sundram@aimst.edu.my; 3 Centre of Excellence for Biomaterials Engineering, AIMST University, Bedong 08100, Kedah, Malaysia; skathir@aimst.edu.my; 4 Faculty of Medicine, Bioscience and Nursing, MAHSA University, Jalan SP 2, Bandar Saujana Putra, Jenjarom 42610, Selangor, Malaysia; drvetriselvan@mahsa.edu.my; 5 Faculty of Applied Science, AIMST University, Bedong 08100, Kedah, Malaysia; 6 Advanced Drug Delivery Laboratory, Faculty of Pharmacy, International Islamic University Malaysia, Kuantan 25200, Pahang Darul Makmur, Malaysia; azad2011iium@gmail.com; 7 Division of Microbiology and NCDs, ICMR-Regional Medical Research Centre, Bhubaneswar 751023, India; swain.shasanksekhar86@gmail.com; 8 Department of Pharmaceutical Chemistry, Faculty of Pharmacy and Health Sciences, Universiti Kuala Lumpur Royal College of Medicine Perak, Ipoh 30450, Perak, Malaysia; mahendransekar@unikl.edu.my; 9 Department of Pharmacognosy, Tishk International University, Erbil 44001, KRG, Iraq; omji.porwal@tiu.edu.iq; 10 Department of Skin & VD, Institute of Medical Sciences and SUM Hospital, Siksha ‘O’ Anusandhan Deemed to be University, Bhubaneswar 751003, India; alakasahoo81@gmail.com; 11 College of Pharmacy, National University of Science and Technology, Muscat 130, Oman; dhanalekshmi@nu.edu.om; 12 Department of Pharmaceutical Sciences, Gurukul Kangri (Deemed to Be University), Haridwar 249404, Uttarakhand, India; vipin@gkv.ac.in; 13 Faculty of Pharmacy, Medicaps University, Indore 453331, MP, India; drsanjay2000jain@gmail.com; 14 Department of Pharmaceutics, NGSM Institute of Pharmaceutical Sciences, NITTE Deemed to be University, Mangalore 575018, India; rnanitte@rediffmail.com

**Keywords:** liposome, ethosome, transethosomes, transfersomes, bioactive

## Abstract

A selected active pharmaceutical ingredient must be incorporated into a cargo carrier in a particular manner so that it achieves its goal. An amalgamation of active pharmaceutical ingredients (APIs) should be conducted in such a manner that it is simple, professional, and more beneficial. Lipids/polymers that are known to be used in nanocarriers for APIs can be transformed into a vesicular formulation, which offers elegant solutions to many problems. Phospholipids with other ingredients, such as ethanol and water, form suitable vesicular carriers for many drugs, overcoming many problems related to poor bioavailability, poor solubility, etc. Ultraflexible liposomes are novel carriers and new frontiers of drug delivery for transdermal systems. Auxiliary advances in vesicular carrier research have been made, enabling polymer-coated ethanolic liposomes to avoid detection by the body’s immune system—specifically, the cells of the reticuloendothelial system. Ultraflexible liposomes act as a cargo system and a nanotherapeutic approach for the transport of therapeutic drugs and bioactive agents. Various applications of liposome derivatives in different diseases are emphasized in this review.

## 1. Introduction

The pertinence of medicinal drugs for the skin is undoubtedly a paradigm as old as humanity; ancient Egypt’s papyrus records portray various medicines for external use. Drug delivery is a blend of art as well as science. Drugs have been used in a variety of ways that reflect pharmacists’ ingenuity and scientific imagination over the centuries. New drug delivery modes have been developed to alleviate the deficiencies of earlier vehicles or, more recently, to refine the delivery of drugs over the course of thousands of years; compounds have been used on the skin as cosmetics, or to treat local conditions [1]. However, most topical compounds and/or medicines are poorly absorbed, if absorbed at all. This is due to the drugs’ molecule size and polarity, as well as the skin’s barrier effect [2].

The topical or transdermal route of drug delivery has numerous advantages over other routes because of patient compliance, preventing the first-pass effect or pre-systemic metabolism of drugs by the liver, with few side effects. However, the major issue with these systems is the transport of therapeutic moieties across the skin due to the tiny pore size of 20–40 nm, which acts as a barrier [2,3]. Topical dermatological dosage forms—such as creams, lotions, and novel carrier-based topical formulations—can be directed to treat skin diseases. By administrating drug molecules to the precise site of action, such as locations of skin diseases, topical formulation products provide the quickest and handiest treatment technique for eczema, psoriasis, wound dressings, oncology-related diseases, and anal fissures. Novel transdermal delivery is necessary to overcome all of the disadvantages of conventional topical/transdermal delivery, and other routes of dosage [4,5]. In the search for safe and powerful treatments, improvements in new medications have, indeed, been a regular practice. It is understood that the issues relating to viability and safety are largely impacted by the delivery of the drug within the biological framework, as there is a considerable deviation from the desired site of activity, i.e., the target site. Dermatology specialists have investigated the utilization of nanotechnology—particularly novel vesicular carrier systems, such as liposomes, and their derivatives transethosomes, transfersomes, ethosomes, and niosomes—as medication conveyance frameworks for topical and transdermal applications [6]. However, the major problem with utilizing the transdermal system is decreased penetration across the skin due to the barrier it presents. Even after improvements in the transdermal delivery systems by nanotechnology or nanocarrier-like liposomes, they still show less penetration through the narrow pores, and mostly reside on the top layer of the skin (i.e., the stratum corneum) due to its rigid structure and non-flexibility. The utilization of elastic vesicles can improve the penetration of large drug molecules compared to conventional liposomes or other topical formulations [7,8,9].

## 2. History of Lipid Carriers and Vesicles

Topical formulations (transdermal) are probably among the most challenging products to develop when it comes to delivering a drug to a specific site [6]. Investigations have been carried out to determine methods by which to intervene in the disease process by supplying therapeutic medicaments to the body at a sustained and controlled rate, with nonstop transport at the site of action, accurate targeting, and low side effects. The transdermal drug delivery system facilitates the dissolution and delivery of a drug across the skin barrier to reach the targeted underlying tissues. The best way to improve drug penetration through different layers of skin is to manipulate the vehicle or to use a drug carrier [10]. For over two decades, analysts have endeavored to ascertain how to utilize the skin as an entryway for medications in order to conquer issues related to conventional methods of drug transportation [7,11,12,13]. A container bearing a formulation needs to be optimized so that the drug carrier bears medicaments in a stable environment with therapeutic efficacy, while maintaining its shelf life during storage [14]. To treat local dermatological disorders, the choice of route is topical skin delivery; issues persist with the conventional topical dosage formulations, as they are not well absorbed into the skin. Topical formulations with a novel carrier system have proven significant for delivering drugs through the skin into deeper tissues, and for achieving higher bioavailability. The scientific voyage of the vesicular carrier began with academic curiosity from a sprinkling of olive oil on saltwater; it ended with egg lecithin smears on glass slides viewed under the microscope [15]. Alec Bangham described the first disclosed bilayer phospholipid systems, earlier called “bangosomes”, now known as liposomes [16]. Gregory Gregoriadis proposed the use of liposomes for drug delivery [17]. The main backbone of liposomes are phospholipids [18]. These biomolecules are also the most critical components of the biological membranes [19,20]. There is intense interest in cutting-edge cargo or delivery systems that are appropriate for the transport of various therapeutic pharmaceutical molecules, specifically being effective in terms of cost, with a lesser side-delivery system that is cost effective, highly effective, and has fewer side effects [21]. Liposomes are a phospholipid bilayer that has been adjusted into a circular arrangement. This circle of molecules encompasses a purge space when controlled appropriately, and it can be filled with several drug molecules. In the face of excess water, phospholipids and other polar amphiphiles form closed concentrated bilayer membranes that trap water and dissolve solutes (e.g., drugs) [22]; their molecular properties and chemical structure control the penetration of a molecule into the skin. For instance, it has been demonstrated that the lipophilicity and hydrogen-bonding capacity have a noteworthy effect on the skin absorption profile [21,22].

Solid lipid nanoparticles (SLNs) are nanostructured lipid carriers (NLCs) that fall in the category of rigid lipid carriers and liposome vesicles, while liquid micelles (LMs) are ultraflexible systems. Vesicular carriers—such as transfersomes, ethosomes, and transethosomes—are the new generation of ultraflexible carriers [23]. SLNs are colloidal carriers, and the purpose of developing these carriers is to overcome the problems related to traditional carriers (such as emulsions, liposomes, and polymeric nanoparticles). SLNs, which are hydrophobic, are exposed to phagocytic uptake by macrophages and targeted by lymph capillaries and lymph nodes, which serve as reservoirs for viruses and other microorganisms [10]. SLNs have gained considerable attention as novel colloidal drug carriers for intravenous applications rather than for transdermal drug delivery [24]. A potential problem in SLNs is the formation of a perfect crystal compared to a dense “brick wall”. The drug load in SLNs is limited due to the construction of the lipid crystal. Crystallization of lipids in SLNs leads to drug expulsion or burst release rather than controlled release. This limitation restricts the use of SLNs in transdermal drug delivery systems [25,26].

Nanostructured lipid carriers (NLCs) are mixed solid and liquid lipids (oils) dispersed in a surfactant-containing aqueous solution. To overcome potential problems with SLNs, NLCs were introduced, aiming to boost drug loading and prevent the expulsion of drugs from SLNs [24,25,26]. The combination of solid and liquid lipids in NLCs allows for enhanced active agent loading with higher stability. Furthermore, improvements have also been accomplished in areas such as skin occlusion, hydration, sun protection, controlled release, and skin targeting. NLCs exhibit superior benefits over other colloidal carriers (e.g., nanoemulsions, polymeric nanoparticles, SLNs), and have been more extensively explored in pharmaceutical technology [27].

Micelles are lipid molecules that are found in aqueous solutions in spherical form; however, they do not have a bilayer or internal cavity. Micelles have attracted attention for the delivery of water-insoluble or partially soluble drugs. Micelles are three-dimensional structures made by the self-assembly of amphiphilic molecules; these are supramolecular, non-static structures formed by surfactant molecules in aqueous solutions [28]. The micelles are formed due to the amphipathic nature of the fatty acids, which means that they contain hydrophobic and hydrophilic groups. They usually have a polar or hydrophilic head group on the surface in contact with water [29]. The hydrophobic or nonpolar tail forms the inner part of the micelles, away from the water’s surface [30]. Micelles are usually spherical structures ranging in size from 2 to 20 nm, based on their composition. Surfactant molecules typically form micelles when the critical micelle concentration (CMC) is reached (the concentration of a surfactant below the CMC is when the surfactant is monomeric in solution, and beyond the CMC, all added surfactants form micelles). Micelles are thermodynamically stable and easily reproducible, but they can be destroyed by water dilution when the surfactant concentration is below its CMC [29,30]. Any formulation to be introduced on the market requires approval from regulatory bodies. Regulatory bodies approve the active ingredient based on the therapeutic application, and excipients become an obstacle for any novel formulation if the excipient has a status of non-acceptance or non-GRAS (generally recognized as safe) materials. Toxicity and the quality of excipients are significant issues for the use of a delivery system. All topical novel formulations, such as the lipid carrier and vesicular systems, must have approved ingredients or GRAS materials [26,27,28,29,30,31]. Most excipients fall in the GRAS category or have been used in marketing products (Table 1) [7,9,13,30,31,32,33,34,35,36,37,38,39,40,41,42,43].

## 3. Ultraflexible Vesicles and Ethanolic Vesicles

**Figure 1:**
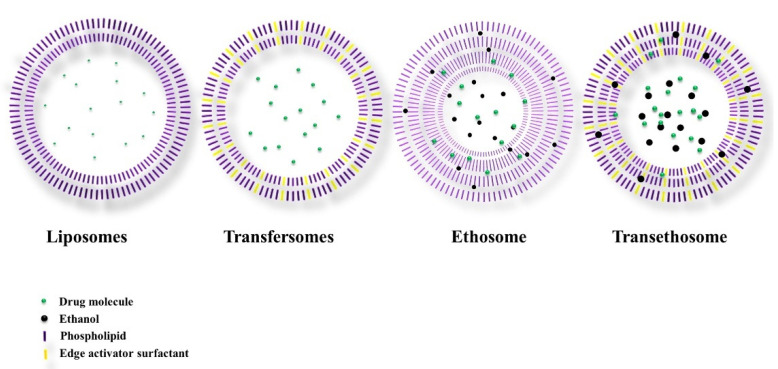
Different vesicular carriers with defined layers and composition.

Nanotechnology in dermatology has dramatically improved traditional liposome compositions in achieving the deeper permeation of active ingredients to different skin strata. Liposomes of ultraflexible vesicles are common vectors in transdermal drug delivery systems that are relatively liquid and deformed [10]. Currently, there are three types of highly flexible liposomes: transfersomes, ethosomes, and transethosomes (Figure 1). In recent years, ultraflexible vesicles have become new liposome carriers with high deformability, high trapping efficiency, a reasonable transdermal drug delivery permeation rate, and suitable transdermal administration [49]. Transfersomes are drug transporters that can permeate intact deep skin. It is believed that the unimpeded passage of these cargo carriers is predicated by two key factors: the high elasticity (ultraflexible or deformability) of the bilayer vesicle, and the reality of an osmotic gradient around the skin. Transfersomes have high surface hydrophilicity, and respond to the gradient of hydration across the dermal tissue [9]; this impels the vesicles through the transcutaneous channels, allowing transfersome vesicles to act as noninvasive drug carriers. Transfersome vesicles have a high degree of flexibility in the bilayer membrane, and show sufficient permeability through the skin. The mechanism behind transfersomes’ penetration is the development of the osmotic gradient generated due to the evaporation of the lipid suspension on the skin’s surface as water evaporates. Transfersomes are strong, intensely deformed bilayers and, therefore, have an increased ability to bind and retain water. Dehydration does not occur in an ultradeformable and highly hydrophilic vesicle; it is not the same as forward osmosis, but it may be related to forward osmosis in the transport process [23]. Ethosomes, developed by Touitou et al., represent the third generation of elastic lipid vesicular carriers. Phospholipids, ethanol, and water are the ethosomal systems. Ethosomes have been reported to improve the supply of different medications to the skin. Ethosomal systems differ from liposomes because the ethanol content of their formulations is relatively high. Ethosomal systems are classified into three classes based on their compositions: classical ethosomes, binary ethosomes, and transethosomes (TEs) [23,44,50,51,52,53]. Song et al. first reported transethosomes in 2012, and the transethosomal system framework contains the fundamental parts of the traditional ethosome, with extra ingredients—for example, permeation enhancers or surface-active agents in their lipid carrier formulation [54]. Transethosomes made of phospholipids, ethanol, water, and an edge activator (surface-active agent) or permeation enhancer have been created. The amalgamation of transfersomes (ultradeformable vesicles) and ethosomes (elastic and flexible vesicles) can cause the vesicular delivery of the drug to go deeper into the skin’s tissue [24,54,55,56,57,58,59,60,61,62,63]. A comparison of all liposome generation vesicles with their mechanisms and compositions is summarized in Table 2 [46,47,48,64]. For a substance to be transdermally absorbed involves some key events, given as follows [2,54,65]:

- Interaction of the substance with the stratum corneum;
- Diffusion of the substance through the stratum corneum;
- Crossing from the lipophilic stratum corneum to the more aqueous viable epidermis;
- Continuing movement from the avascular epidermis to the highly perfused dermal tissue;
- Uptake through the microcirculation to the systemic circulation.

**Figure 2:**
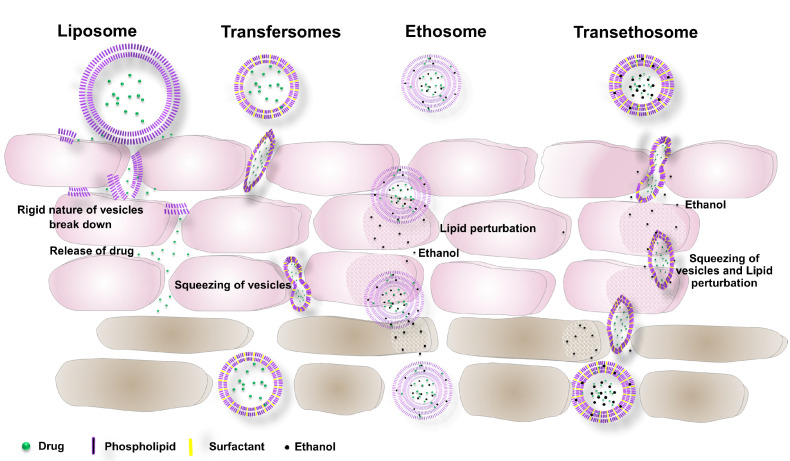
Mechanism of the vesicular carrier crossing the stratum corneum of the skin.

**Figure 3:**
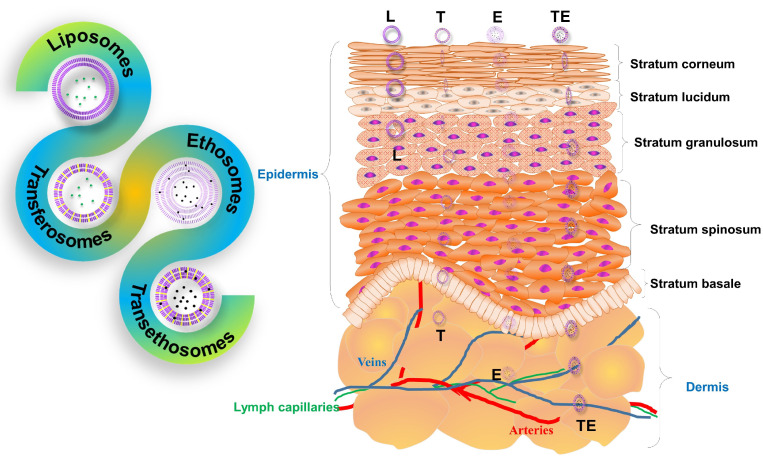
Different vesicular carriers with different penetration capabilities (L: liposome; T: transfersome; E: ethosome; TE: transethosome).

Two mechanisms were reported: (1) elastomechanics, and (2) a gradient of transepidermal water activity (Figure 2 and Figure 3). When the substance has to pass the stratum corneum, it usually has two pathways in humans: (a) transcellular, and (b) intercellular; the main corridor is the intercellular pathway between the corneocytes, which insinuates that the stratum corneum lipids play a crucial role in the function of the skin barrier [57]. However, appendices and other diffusion shunts can also play a significant role for very lipophilic and large molecules (and a few electrolytes). A skin-penetrating chemical must first pass the highly vascularized lipid membrane structure (SC—stratum corneum) and proceed to the blood vessels after a more watery layer (lower epidermis and dermis). The substance’s permeability coefficient tends to increase with its lipophilicity. Penetration of the SC corneocytes’ penetration is the primary way through which most of the molecules penetrate the skin [48,56,57]. Liposomes, due to the presence of cholesterol, have a rigid nature, and they are unable to cross deeper layers of skin. Liposome vesicles break down due to the rigidness in the upper layers of the skin, and release drugs only in the epidermis layers [66,67]. Transfersomes that can be loaded with a therapeutically active moiety can overcome the skin barrier spontaneously to permeate the drug into the deep tissue layers of the skin, as it is drawn from the dry surface to the water-rich region beneath the skin. When transfersomes are applied to human skin, the transfersome carrier seeks and finds hydrophilic channels or “pores” between the cells of the skin or corneocytes, which open up wide enough for the complete vesicle to carry through its drug payload without compromising its vesicular structure (flexibility) or releasing the payload. In order to deposit the payload at different layers of the skin, the carrier then avoids the local microvasculature when the active component is preferentially released to the targeted tissue. The depth and rate of the drug deposition are determined mainly by the nature of the ingredients used in the transfersomes [68]. Ethosomes are ethanolic and noninvasive carriers that allow the active ingredient to penetrate deep layers of the skin or enter systemic circulation. Ethanol is a well-known permeation enhancer that gives ethosomes distinctive properties, such as strong flexibility and deformability, allowing them to penetrate deeply through the skin and improve drug penetration and deposition [69]. The hydrophobic nature of ethanol’s carbon tail makes it much easier to penetrate the hydrophobic chain areas of lipid bilayers and ethanol molecules condensing close to the interface area between lipids and the surrounding waters—that is, near the interface region, there is a sharp increase in ethanol’s density. The addition of ethanol also has an apparent negative effect on the surface of ethosomes, improving their stability because of electrostatic repulsion. Ethanol perturbs the lipids in the skin layers and helps the vesicular carrier to penetrate deeper into the tissue of the skin [70]. Transethosomes penetrate the skin layer via a combination of the transepidermal osmotic gradient and the effect of ethanol [71].

## 4. Liposome Vesicle Generation

### 4.1. Transfersomes

The development of an osmotic gradient is the mechanism behind the penetration of the transfersomes, as when a lipid suspension is applied to the skin’s surface, water evaporates. Transfersomes show strong deformability of the bilayer, and have increased ability to bind and retain water [24,59]. The crystal arrangement of lipid molecules in the stratum corneum is altered by penetrated surfactants alongside keratinocytes. Increased lipid molecule fluidity improves the penetration of water-soluble molecules. Ultraflexibility enables transfersomes to squeeze through small pore channels, driven by the gradient of the skin’s water activity, while the hydrophilic surface facilitates the loosening of hydrophilic gaps in the skin. Since transfersomes tend to avoid a dry environment, they are attracted to higher layers of water in skin tissue, leading to the spontaneous migration of the drug-loaded vesicles through the skin barrier [24,60,61,62]. When applied on the (non-occluded) skin surface, they penetrate the skin barrier and try to reach the deeper strata (water-rich portion), where they are properly hydrated. Then, as a result of natural transepidermal activity, they travel deeper into the epidermal layer via dehydration of the lipid vesicles in the stratum corneum. The lipid-to-surfactant ratio significantly affects the flexibility of the bilayer of transfersome carriers. Transfersomes can act as both drug carriers and enhancers of penetration [25,26,27,28,29]. Barrier penetration involves reversible bilayer deformation, but must not jeopardize the integrity of the vesicle or the barrier structures, so as to maintain an unimpaired hydration affinity and gradient. Transfersome absorption is, therefore, a function of the hydration gradient found throughout the epidermis, stratum corneum, and atmosphere [46,61,62].

### 4.2. Ethosomes

Ethanol interrelates with the lipid materials in the polar head area, bringing about a decrease in the changing temperature of the lipids in the stratum corneum, increasing the ease of their passage and diminishing the multilayer lipid’s compactness. This is preceded by an ethosome effect, which encompasses lipid penetration and permeation by creating new pathways via the malleability of ethosomes and their fusion with skin lipids, which leads to drug releases from vesicles into the dermis and the deep layers of the skin (Figure 2). Ethanol can also provide vesicles with soft, highly flexible characteristics so as to permeate the skin’s dermis layers easily and quickly [2,46,47,48,64].

### 4.3. Transethosomes

The combination of ethosomes with a penetration enhancer allows vesicles to penetrate deeper skin tissues and release the drug into systemic circulation, demonstrating a systemic effect. Oleic acid is a permeation enhancer that improves the permeation of therapeutic drugs into the skin by acting on a cornified SC lipid envelope [2]. Ceramides are the main lipid in the SC lamellar sheet. Oleic acid supports the separation of the phase by reducing the Tm of ceramides. Another penetration enhancer, propylene glycol (known for its multiple uses in topical/transdermal formulations), prompts the interaction of propylene glycol and keratin in the SC, which causes SC corneocyte disturbances and improved drug penetration. In these ways, propylene glycol shows a penetration-enhancing effect [72]. Adding permeation enhancers to the ethosome leads to transethosomes. Ethanol and propylene glycol/oleic acid act as dual penetration ingredients in transethosomes. Ethanol acts on the corneodesmosomes that fluidize lipids and loosen the corneocytes for easier access to skin layers, whereas penetration enhancers (surfactant or miscellaneous agents) that act on the keratin present in the corneocyte cells loosen the dense protein structure, making skin more permeable for drugs and vesicles [2,46,47,48,64,72,73,74,75].

**Table 2:** Differences between liposomes, transfersomes, ethosomes, and transethosomes.

Characters	Liposomes	Transfersomes	Ethosomes	Transethosomes	References
Vesicles	Bilayer lipid vesicle	2nd generation elastic lipid vesicle carriers	3rd generation elastic lipid vesicle carriers	3rd generation	[2,24,32]
Lamellarity	Uni/bilayer lipid vesicle	Double bilayer lipid vesicle	Multiple bilayer lipid vesicle	Multiple bilayer lipid vesicle	
Composition	Phospholipids and cholesterol	Phospholipids and edge activator surfactant	Phospholipids and ethanol	Phospholipids, edge activator surfactant, and ethanol	[2,24,32,42,53,54,56,76]
Surfactant role	Phospholipid (lecithin)	Sodium deoxycholate	Phospholipid (lecithin) and ethanol	Sodium deoxycholate, oleic acid	
Characteristics	Microscopic spheres (vesicles)	Ultraflexible liposome	Elastic liposome	Ultraflexible elastic liposome	[2,24,32,42,53,54,56,76]
Flexibility	Rigid in nature	High deformability due to the surfactant	High deformability and elasticity due to the ethanol	Ultra-deformability due to the surfactant and ethanol	[2,53,54,56,76]
Permeation mechanism	Diffusion/fusion/lipolysis	Deformation of vesicle	Lipid perturbation	Lipid perturbation due to the ethanol and deformation of vesicles by surfactant	[2,24,32,56,76]
ζ potential	Neutral	Positive or negative	Negative	Positive or negative	
Extent of skin penetration	The penetration rate is significantly lower, as the stiff shape and size do not allow it to pass through the stratum corneum	Can easily penetrate through paracellular space due to the flexible structure	Can easily penetrate through paracellular space via ethanol effect	Can easily penetrate through paracellular space via flexible structure and ethanol effect	[2,24,32,42,53,54,56,76]
Route of administration	Oral, parenteral, topical, and transdermal	Topical and transdermal	Topical and transdermal	Topical and transdermal	[2,24,32,42,53,54,56,76]
Limitations	It cannot penetrate into deeper skin	Due to the surfactant, it may cause skin irritation and stable in gel form only	All drugs are non-soluble in ethanol	Due to the surfactant, it may cause skin irritation, and drug loss during the process of formulation	[2,24,32,42,53,54,56,76]
Marketed products	AmBisome, DaunoXome Doxil, Abelcet	Transfersomes^®^ (Idea AG) Flexiseq	Nanominox, Cellutight EF, Noicellex, Decorin Cream	Nil	[2,24,32,42,53,54,56,76]

## 5. Application of Ultraflexible and Elastic Vesicles

Ultraflexible and elastic vesicles have been explored for many therapeutic drugs used to treat disease. These vesicles are used as topical and transdermal delivery systems containing drugs, with different means of releasing the drugs to the skin for local or systemic effect. Different drugs with ultraflexible and elastic vesicles are listed in Table 3.

### 5.1. Applications of Ultraflexible and Elastic Vesicles in Cancer

Gupta et al. engineered a pro-transfersome-bearing cisplatin delivery system for cutaneous epithelial malignancies. The system’s in vivo performance results showed an increase in the drug’s therapeutic efficacy, with reduced systemic toxicity. Pro-transfersome is a solid form of transfersome that must be dispersed in an aqueous vehicle in order to form transfersome vesicles [82]. Elastic vesicles can penetrate the stratum corneum’s microscopic pores under the effect of transcutaneous hydration (osmotic gradient) caused by differences in the concentration of water between the epidermis and the interior of the skin. Formulations typically contain short-chain alkanols that increase the penetration of drugs into the stratum corneum by dramatically increasing its fluidity [76]. The presence of a fluorescence molecule in the deeper skin layer shows that the pro-transfersome formulation has better skin permeation ability. The system’s ultraflexible nature appears to allow better drug delivery at the tumor site [76,82].

Novel hyaluronic acid (HA)-modified transfersomes deliver drugs to lymphatics via the transdermal route for tumor metastasis therapy. HA–GMS–T (hyaluronic acid– glycerol monostearate–transfersome)-loaded doxorubicin was able to penetrate the deep skin tissue efficiently, leading to increased lymphatic absorption. Above all, hyaluronic acid effectively improved the absorption of drug-loaded nanocarriers by tumor cells. Since transfersomes tend to avoid a dry environment, they are attracted by higher layers of water in skin tissue, leading to a serendipitous migration of the drug-packed vesicles through the different layers of the skin barrier. Based on the vesicle’s size and entrapping efficiency, the results showed that Tween-80 appears to be a better edge activator than Span-80. The transfersome gel improved the skin permeation and skin deposition of 5-FU in vitro compared to the market formula [61,83].

The ethosomal system is used to deliver anticancer drugs for skin cancers. The use of paclitaxel-bearing ethosomes in the skin enhanced the penetration of paclitaxel in the membrane model of the stratum corneum–epidermis, and increased its antineoplastic activity in the model of squamous-cell carcinoma, particularly in comparison to the free drug suspension [7,35]. The alcohol in the ethosome initiates transdermal penetration and drug release, with the effect of improving penetration. Ethosomal mitoxantrone (MTO) is not applied directly on the skin as a transdermal formulation; instead, it has been converted into a viscous gel that can reside for a long time and permeate deeper tissue. The ethosome-embedded Hydroxypropyl methylcellulose (HPMC) gel has sufficient viscosity to adhere to the skin’s surface and increase the residence time. Transdermal MTO illuminates the hope of efficacious and affordable chemotherapy for melanoma. MTO alone cannot penetrate deeper tissues of skin when compared to MTO ethosomal gel, which is an effective therapeutic strategy for noninvasive melanoma, without the debilitating side effects of intravenous anticancer injection [84].

**Figure 4:**
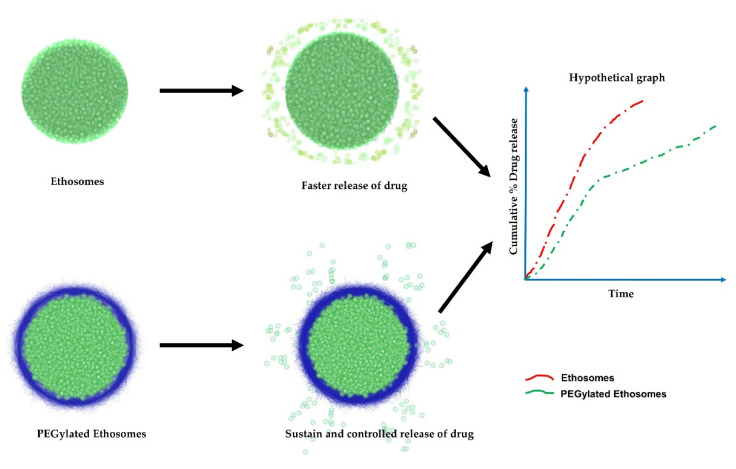
Schematic and hypothetical diagram of PEG ethosome and ethosome release kinetics.

Otsuka et al. also found that polyethylene glycol (PEG) can enhance stability and, consequently, increase drug delivery to the tumor, improving therapeutic efficacy [85]. Auxiliary advances in vesicular carrier research have been conducted, allowing polymer-coated ethanolic liposomes to avoid detection by the immune system—specifically, the cells of the reticuloendothelial system (RES) [85]. PEG–ethosomal formulations bearing paclitaxel had a release rate that gradually decreased due to the presence of PEG. In the first 3 h, the PEG–ethosomal formulations showed rapid release during the initial hours and time-lapse, and the drug was released at a sustained and controlled rate due to presence of PEG, which had swollen and formed a barrier against the release of paclitaxel [74,84,85,86]. PEG ethosomes and ethosome release kinetics are illustrated in Figure 4.

Fisetin, a natural flavonoid, inhibits various cancer cells’ propagation. The bioavailability of fisetin was enhanced when transethosome carriers were used. Thermo-analytical techniques have shown that the formulation of transethosome vesicles fluidizes the rigid membrane of rats’ skin for smoother penetration of fisetin transethosomes. Transethosome vesicle formulations were found to be a potentially useful drug carrier for the dermal delivery of fisetin, and were able to permeate deeper skin [87,88].

### 5.2. Fungal Infection

Miconazole nitrate is a topical antifungal agent that is used to treat topical superficial fungus on the skin. Due to its low permeation capability, there is a need for a carrier that squeezes through the skin barrier and permeates the skin layers, showing maximum efficacy. Transfersome technology was used to develop vesicular carriers bearing miconazole nitrate, and compare them with the marketed product Daktarin. It should be noted that antifungal activity in transfersome gels is much higher than Daktarin, based on minimum inhibitory concentration (MIC) results. Transfersome gels can cross the stratum corneum barrier as a result of their deformability capability, releasing the drug in a sustained manner [41].

Ethosome-bearing fluconazole was prepared using a lipid thin-film hydration method, and microscopic images showed a spherical multilamellar structure. Ethosomal formulations showed higher release kinetics than liposome and hydro–alcoholic solutions of fluconazole. Thirty percent ethanol in ethosomes was the optimal formulation in terms of drug diffusivity, and a further increase in ethanol led to a decrease in drug diffusivity. The in vivo antifungal activities of the drug fluconazole encapsulated in liposomes, ethosomes, and marketed formulations were compared. The ethosomal formulation showed higher antifungal activity than any other formulation, and it improved disease remission and reduced the duration of treatment [89].

Econazole nitrate (ECN) is a topical antimycotic and antifungal agent, which is used against fungal growth on the skin. Transethosomes bearing econazole nitrate were prepared by the homogenization method. Econazole-nitrate-loaded transethosome formulations effectively deliver econazole nitrate transdermally, in a controlled and sustainable manner, for the effective eradication of cutaneous fungal candidiasis. A transethosome formulation was compared with the branded product Ecoderm. The results revealed that the transethosome release follows zero-order kinetics, with high drug retention in the skin, and more potent activity against fungus than the marketed product [90].

### 5.3. Inflammation

Significant topical anti-inflammatory effects suggest that flexible or elastic vesicles loaded with curcumin (transfersomes) can relieve rheumatism and provide the additional benefit of suppressing inflammatory responses from tissue injury. Elastic vesicles (EVs) have been deemed to be the best choice for the delivery of topical curcumin. Vesicular systems loaded with curcumin have shown improved skin permeability of curcumin and protection against degradation. Vishnu Industrial Chemicals Company (VICCO) turmeric skin cream and elastic vesicles bearing curcumin were compared in terms of flux value and skin retention of curcumin; the VICCO cream performed considerably worse than elastic vesicles [7,91].

Sinomenine hydrochloride (SH) is an alkaloid anti-inflammatory drug with low oral bioavailability and increased gastrointestinal (GI) side effects. The issues can be resolved by entrapping the drug in an ethosome formulation, which is safe and effective in anti-inflammatory treatment, enhancing the drug’s bioavailability. Sinomenine has a short half-life, and increased gastrointestinal (GIT) side effects. The drug is loaded in an ethosome formulation, which reduces skin irritation and increases the drug’s half-life. Ethosomal formulations are a good vehicle for the transdermal delivery of SH, and a possible remedy for treating local inflammation [92].

Gout is an inflammatory arthritis disease, and colchicine is used for its treatment; its oral administration has poor bioavailability, extensive first-pass metabolism by liver, and severe side effects on the gastrointestinal tract. When colchicine was orally administered, it led to GIT side effects due to its low therapeutic index. The deposition of colchicine in the body causes a clampdown on bone marrow. The transethosomal of colchicine, when applied to the skin, permeates deeper skin layers and bypasses the cutaneous blood circulation, because the size of the vesicular carrier is sufficient to avoid entering the blood’s circulation. Instead, they cross over to the subcutaneous capillary bed, and reach the subcutaneous tissues. The vesicular carrier acts as a depot system, slowly releasing the colchicine to the affected area and sustaining the release of the drug locally. Therefore, the side effects of colchicine on other bodily organs are prevented due to local drug release by transethosomes. The colchicine released from the transethosomes acts on phagocytes, inhibiting their activity, and inhibits leukocyte migration to the joints, preventing the growth of other urate crystals in the joints [93]. Transethosomes bearing colchicine (TEs) were prepared to overcome the issues of drug metabolism by the liver, as transfersomes preserve the encapsulated drug against metabolic degradation. The drug entering directly into the subcutaneous tissue prevents the metabolism of colchicine by the liver and enhances the bioavailability of colchicine to the targeted site, in a dose-dependent manner. Ex vivo skin penetration studies have shown that transethosomal gels have greater skin permeation properties than non-ethosomal (NE) gel formulations. The transethosome formulation bearing colchicine for transdermal application is a potential drug cargo for gout treatment—a substitute for the oral route for colchicine [75].

### 5.4. Analgesic

Diclofenac is an analgesic drug that relieves the chronic pain of different pain-related disorders via topical or transdermal application. Diclofenac’s conventional formulation cannot penetrate the drug to deeper tissues of the skin, which is required in order to relieve pain. Transfersomes are an ultraflexible vesicular carrier that can squeeze into the stratum corneum’s smallest pores (adjacent space between coenocytes) [94]. Transfersomes that are deformable and have high flexibility can reach deeper skin layers (dermis), leading to systemic circulation. These vesicles have more penetration power compared to classical liposomes. Vesicular transfersome dispersion and vesicular transfersome gel can permeate the skin layers and deposit diclofenac sodium at a rate 2–3-fold that of classical liposomes. Transfersomal formulations can act as a reservoir for drugs in the skin and increase diclofenac’s pharmacological effects [94,95].

Aceclofenac is a potent analgesic drug, and the ethosomal system is a promising carrier for its transdermal delivery, helping to reduce the dose of topically applied medications. The ethosomal formulation suggests that systemic treatment of osteoarthritis and rheumatoid arthritis can be replaced by local treatment, which would lead to a reduction in gastrointestinal side effects [72].

In rheumatoid arthritis, osteoarthritis, and other inflammatory diseases, piroxicam is used as an analgesic and anti-inflammatory drug. It has been reported that ulcerative colitis and gastrointestinal irritation are caused by its oral administration. An alternative or substitute route is required in order to overcome the side effects caused by the oral administration of piroxicam. Transdermal delivery of transethosomes and other vesicular formulations bearing piroxicam were prepared via the thin-film hydration method. Drugs were released from transethosomes via the diffusion mechanism. The results show that the transethosome formulation in the gel form is superior to other vesicular systems [96,97].

### 5.5. Viral Infection

Transfersomes (flexible liposomes) may be favorable cargo for the transdermal delivery of acyclovir sodium. The transfersome (ultraflexible liposomal) formulation of acyclovir sodium has shown a better transdermal delivery profile compared to classical liposomes. The flexibility of these vesicles is due to the simultaneous presence of different stabilizing molecules (phospholipids) and destabilizing molecules (surfactants), and their tendency to reorganize in bilayers [98].

Stavudine is an antiretroviral drug used in the treatment of human immunodeficiency virus (HIV). Ethosomes can increase the transdermal flux, prolong the drug release, and present an attractive route for the sustained delivery of stavudine. Ethanol offers a negative charge to vesicles and prevents their accumulation. The addition of ethanol to ethosomal formulations provides negative charges. Lipid perturbation, along with the elasticity of ethosome vesicles, seems to be the main contributor to improved skin permeation [99,100].

Ethosomes with penetration enhancers (surfactant) are transethosomes. Transethosome formulations bearing lamivudine, containing phospholipids, ethanol, and a permeation enhancer (propyl glycol or oleic acid), have shown deeper penetration of drugs into skin tissues. Permeation enhancers in transethosomes—such as propylene glycol—act by intermingling with the keratin protein present in lipid-depleted corneocytes, while oleic acid endorses phase separation by decreasing the melting temperature of ceramides; formulations made using this permeation enhancer have shown promising results in terms of drug permeation of the skin. Transethosomes with oleic acid have shown faster penetration or a reduction in lag time [2,46,47,48]. Incorporation of dicetyl phosphate in ethosomes exhibits the deeper penetration of the drug/marker lamivudine in rat skin, while varying the concertation of dicetyl phosphate shows different levels of penetration of the drug/marker in the skin. The negative charge of the dicetyl phosphate in ethosomes is the driving force for the deeper penetration of the drug/marker into the skin layers [48].

### 5.6. Bacterial Infection

Efficient delivery of antibiotics/antibacterial materials into deep skin strata from ethosomal applications could be highly beneficial, and can reduce possible side effects and other drawbacks associated with systemic treatment [101,102,103]. Ethosomes are efficient carriers for the delivery of erythromycin to bacteria localized within the deep skin strata in order to eradicate staphylococcal infections [83]. Both molecules—the phospholipid, and the antibiotic—were delivered from the ethosome to a maximum possible depth of 200 µm (dermatome skin), and precise localization of the two molecules was observed in the skin [103,104].

The antibiotic neomycin is used to prevent or treat bacterial skin infections. Transfersomes are a form of a flexible or ultradeformable vesicle bearing surfactants and phospholipids. Neomycin is a BCS-III class drug, with high solubility and a poor permeation rate; this issue can be resolved by incorporating it into transfersomes [102]. Neomycin transfersomes cross the stratum corneum via transepidermal osmotic gradient and the edge activator effect, release the drug into the different layers of skin, and improve the permeation of the drugs into the skin.

Caffeine is a powerful stimulant and antibacterial against *P. aeruginosa*. Transethosomes loaded with caffeine have been developed, and transethosomes are the most deformable vesicles, easily changing their conformation, and allowing a higher membrane passage. Transethosomes act as cargo vehicles for the delivery of caffeine, permeate the stratum corneum via the osmotic gradient and the ethanol effect, and penetrate the different layers of skin, eradicating the *P. aeruginosa* skin infection [105].

### 5.7. Other Disease

Capsaicin is the active ingredient of capsicum; it has the ability to relieve muscle or joint pain when applied topically. With capsaicin, in terms of its penetration power to reach the site of action, due to the skin barrier, it can cause an irritant effect when applied to the skin. Transfersome formulation of capsaicin was compared with a marketed product (Thermagel). Both systems had the same dose, but Thermagel showed a lower penetration power to reach the deeper skin tissue than the transfersome formulation, due to the transepidermal osmotic gradient and edge activator effect. Owing to its formulation in the transfersomal system, the irritant effect of capsaicin was reduced, making transfersomes the formulation of choice for anti-arthritis agents [106].

Hyaluronic-acid-coupled propylene-glycol-based ethosomes (HA–PG–ES) bearing curcumin were developed to treat psoriasis. Hyaluronic-acid-coupled propylene glycol has been used to target highly expressed CD44 in psoriasis. HA–PG–ES containing curcumin is a state-of-the-art strategy to treat psoriasis [107].

Curcumin is the active ingredient of turmeric, which is yellowish-orange in color; it has valuable therapeutic properties. It also has certain limitations, such as poor solubility and poor penetration power when applied to the skin. Transethosomes were prepared with the composition of a phospholipid, ethanol, and a surface-active agent (Span 80). A transethosome gel of curcumin was developed, and was found to overcome the limitations of curcumin [108].

## 6. Future Perspectives

The field of nanotechnology in medicine, in terms of vesicular delivery systems, will provide several themes and solutions for addressing healthcare challenges in the coming decades. The design and architecture of a topical or transdermal formulation combine artistic skills with scientific knowledge of excipients, physical properties of the formulations, skin physiology, and formulation dynamics. Because of their excellent tolerability and performance, these vesicular systems have a wide range of potential therapeutic applications. Elastic, flexible, and deformable vesicles are advanced liposome vesicles, which act as cargo for many therapeutic agents and address many diseases. The variability of kinetic releases from vesicular carriers makes them future novel delivery systems. This drug transporter cargo vesicular system carries drugs ranging from hydrophilic to hydrophobic. Ultraflexible liposomes are mainly focused on using various methods (e.g., PEGylation, biotinylation) for cellular targeting. Scientists all around the globe are continuing to work on strengthening this vesicular carrier system by making it more stable in nature, so as to avoid content leaching, oxidation of lipids, and uptake by biological defense systems. Improvements in the transport of bioactive molecules through the skin by ultraflexible liposomes create new opportunities for the development of effective therapies.

## Figures and Tables

**Table 1:** Different common ingredients are used for the preparation of the vesicular systems for topical or transdermal application.

Type	Composition	Characteristics	Preparation Method	References
Micelles	Block–Polymer Chitosan grafted with palmitoyl Poly(D,L-lactide)-b-methoxy-PEG Poly(β-benzyl-L-aspartate)-b-PEG PEG-b-poly(lactic-co-glycolic acid) (cyclic RGD peptide-modified) Surfactant Pluronics	Monolayer vesicles forming agent Permeation enhancer and to improve stability	Simple dissolution Dialysis Oil-in-water emulsion Solvent evaporation Lyophilization	[7,28,29,30,38,44,45]
Liposomes	Phospholipids 1,2-Dipalmitoyl-sn-glycero3-phospocholine monohydrate Soya phosphatidylcholine Egg L-α phosphatidylcholine Dipalmitoylphosphatidylcholine Hydrogenated lysolecithin Stabilizer Cholesterol Polyethylene glycol Organic Solution Chloroform Isopropyl alcohol Ethanol Buffering Agent PBS buffer 7.4 pH	Bilayer vesicles forming agent Rigidity and stabilizer to bilayer vesicle As a solvent As a hydration medium	Rotary flask evaporation—sonication method Thin-film hydration technique Sonication method French press cell method Freeze–thaw method	[15,19,20,22,31]
Ethosome	Phospholipids Soya phosphatidylcholine DPPG (1,2-dipalmitoyl-sn-glycero-3-phosphatidylglycerol) Egg L-α phosphatidylcholine Organic Solution Ethanol (20–45%) Isopropyl alcohol Stabilizer Propylene glycol Cholesterol Dicetyl phosphate/Stearylamine Permeation Enhancer Propylene glycol Ethanol Tween 80 Span 80 Buffering Agent PBS buffer 7.4 pH	Bilayer vesicles forming agent, the base of the vesicles of which are biocompatible As a solvent and fluidized the bilayer, ethanol can induce hydrocarbon inter-digitation and increases inter-membrane separation in the gel phase For providing stability to vesicle membrane and entrapment efficiency of drugs, prevent aggregation of the vesicles For providing the softness for vesicle membrane and as a penetration enhancer As a hydration medium	Classical cold method Hot method Ethanol injection Sonication method Thin-film hydration method Reverse-phase evaporation method Transmembrane pH-gradient method	[2,46,47,48]
Transfersomes	Phospholipids Soya phosphatidylcholine Lecithin Egg L-α phosphatidylcholine Edge Activator Surfactant Sodium deoxycholate Sodium cholate Sodium oleate Tween 80 Span 80 Organic solution Ethanol Methanol Buffering Agent PBS buffer 7.4 pH	Vesicles forming ingredient, and with the combination of edge activator it forms the flexible bilayer Vesicles forming components and deforming agent and flexibility producing ingredient; they act as permeation enhancers As a solvent medium As the hydrating agent and aqueous medium	Rotary flask Evaporation–sonication method Thin-film hydration technique Ethanol injection method	[2,4,9,41,42,43]
Transethosomes	Phospholipids Soya phosphatidylcholine Dipalmitoyl phosphatidylcholine Distearoyl phosphatidylcholine Egg L-α phosphatidylcholine Organic Solution Ethanol Isopropyl alcohol Surfactant Sodium deoxycholate Sodium cholate Sodium oleate Linoleic acid Oleic acid Stabilizer Propylene glycol Cholesterol Permeation Enhancer Propylene glycol Ethanol Tween 80 Span 80 Buffering Agent PBS buffer 7.4 pH	Bilayer vesicles forming agent, base of the vesicles which are biocompatible As a solvent and fluidized the bilayer of vesicles Deforming agent and flexibility producing ingredient, they act as a permeation enhancer Vesicles forming components and deforming agent and flexibility producing ingredient For providing the stability to the vesicle membrane As a hydrating medium	Classical cold method Hot method Ethanol injection Sonication method Thin-film hydration method Reverse-phase evaporation method	[2,4,35,43]

**Table 3:** Recent research on ultraflexible liposome generation systems.

Vesicular Carrier-Based Dosage Form	Drug	Category of Drug	Dosage Form	Disease	Outcomes	Year/Reference
Ethosome and transethosome	Mangiferin	Antioxidant and anti-inflammatory	Transdermal delivery system	Skin disorders related to pollutants. Potent carcinogens: cigarette smoke can cause melanoma, atopic dermatitis, and eczema.	Ethosomal approach offers a new delivery system for targeted delivery of mangiferin.	2021 [77]
Ethosome-based hydrogel	Carvedilol	Anti-hypertensive	Topical delivery	Hypertension	Improved percutaneous permeation and sustained release of carvedilol, and increases in bioavailability.	2021 [70]
Binary ethosomes	Fisetin	Anticancer	Transdermal delivery system	Cancer	Alternative dosage forms for the management of skin cancer; fluidized the rigid membrane of the skin of rats for smoother penetration of fisetin transethosomes.	2019 [34]
Ethosome gel	Carvedilol	Anti-hypertensive	Transdermal delivery system	Hypertensive angina pectoris	Enhances skin permeation of drugs and reaches into the systemic circulation.	2019 [35]
Liposomes, ethosomes, and transfersomes: nanovesicular hydrogels	Diflunisal	Anti-inflammatory Anti-nociceptive analgesic	Transdermal delivery system	Inflammatory diseases	Deeper penetration of drugs into different layers of skin; shows systemic circulation.	2019 [7]
Polyethyleneimine and sodium-cholate-modified ethosomes	Doxorubicin and curcumin	Anticancer	Transdermal delivery system	Melanoma	A combination of cytotoxic agents and chemosensitizers as well as nanocarriers can help to overcome multidrug resistance (MDR) of cancer.	2019 [36]
Ethosomes and lipid-coated chitosan	Ferrous chlorophyllin	Anticancer	Transdermal delivery system	Squamous-cell carcinoma	Potential for the treatment of SCC using PDT. A higher PDT effect was observed both quantitatively and qualitatively with PC/CHI in SCC monolayers and 3D spheroids compared to ethosomes.	2019 [37]
Ethosome and liposomes	Rosmarinic acid (RA)	Anti-aging, antioxidant, anti-collagenase, anti-elastase	Transdermal delivery system	Protects against free radicals and reduces wrinkles; inhibition of elastase enzyme	Ethosome and liposomes improved and increased RA skin penetration significantly.	2019 [39]
Ethosomal hydrogel	Resveratrol	Anti-aging, anti-proliferative, anti-inflammatory	Transdermal delivery system	Extrinsic skin ageing, psoriasis	Enhanced skin permeation and retention; permeation flux, and skin deposition were also found to be improved compared to conventional cream.	2019 [13]
Microneedle- loaded ethosome	Paeoniflorin	Anti-inflammatory	Transdermal delivery system	Rheumatoid arthritis	Combination of technology to enhance the penetration of paeoniflorin.	2018 [40]
Transfersomal gel	Miconazole nitrate	Antifungal	Transdermal delivery system	Superficial fungal infections, Candida skin infections	Transfersomal gel showed higher antifungal activity than marketed conventional formulation.	2018 [41]
Polyamido amine dendrimer G3 transfersomal gel	Lidocaine	Local anesthetics	Topical delivery	Reduces pain from sunburns and insect bites	Permeation and localization of drug in skin tissue. Prolongs the activity of lidocaine in a sustained manner.	2019 [9]
Ethanol-based malleable liposomes	Cytarabine	Anticancer	Transdermal delivery system	Acute myeloid leukemia	Alternative delivery of drugs for the treatment of leukemia, with low side effects and sustained release.	2018 [42]
Vesicles with a physical method combination	Calcein	Fluorescent dye	Transdermal delivery system		Amalgamations of ethosomes and electroporation or a physical sonoporation method to enhance the model drug’s penetration into skin layers.	2018 [43]
Ethosomes and transfersomes	Sulforaphane	Anticancer and antiproliferative	Transdermal delivery system	Melanoma	Both vesicle types have enhanced percutaneous permeation.	2019 [78]
Ethosomes	AgNPs and sericin	Anticancer	Transdermal delivery system	Non-melanoma skin carcinoma	Novel loom for treatment of non-melanoma skin carcinoma (NMSC).	2021 [79]
Transethosome	Brucine and strychnine	Anticancer	Transdermal delivery system	liver cancer	Transethosome targets the hepatoma cells and sustains the release of drugs for a prolonged time to prevent the proliferation of cells.	2021 [71]
Binary ethosome	Aprepitant	Morpholine-based antiemetic	Transdermal delivery system	Highly emetogenic chemotherapy	Binary ethosome improved the permeation of the biopharmaceutics classification system BCS-IV drug aprepitant into the skin, compared to suspension.	2021 [80]
Ethosomes	Celecoxib and paclitaxel	Nonsteroidal anti-inflammatory and anticancer	Transdermal delivery system	Inflammation and cancer	Both drugs’ solubility was enhanced, and their penetration into skin was improved by ethosome carriers.	2020 [81]

## Data Availability

Not applicable.

## Abbreviations

API	Active pharmaceutical ingredient
RES	Reticuloendothelial system
NLCs	Nanostructured lipid carriers
GRAS	Generally recognized as safe
TEs	Transethosomes
SC	Stratum corneum
HA	Novel hyaluronic acid
5-FU	5-Fluorouracil
HA-GMS-T	Hyaluronic acid–glycerol monostearate–transfersome
MTO	Mitoxantrone
PEG	Polyethylene glycol
MIC	Minimum inhibitory concentration
ECN	Econazole nitrate
EVs	Elastic vesicles
VICCO	Vishnu Industrial Chemical Company
GIT	Gastrointestinal tract
SH	Sinomenine hydrochloride
NE	Non-ethosomal
HIV	Human immunodeficiency virus
HA–PG–ES	Hyaluronic-acid-coupled propylene-glycol-based ethosome
SLN	Solid lipid nanoparticles
PDT	Photodynamic therapy
SCC	Squamous-cell carcinoma
